# Combined effect of obesity and diabetes on early breast cancer outcome: a prospective observational study

**DOI:** 10.18632/oncotarget.22977

**Published:** 2017-12-05

**Authors:** Giuseppe Buono, Anna Crispo, Mario Giuliano, Carmine De Angelis, Francesco Schettini, Valeria Forestieri, Rossella Lauria, Matilde Pensabene, Michelino De Laurentiis, Livia Silvia Adriana Augustin, Alfonso Amore, Massimiliano D'Aiuto, Raffaele Tortoriello, Antonello Accurso, Ernesta Cavalcanti, Gerardo Botti, Maurizio Montella, Sabino De Placido, Grazia Arpino

**Affiliations:** ^1^ Department of Clinical Medicine and Surgery, Oncology Division, University of Naples Federico II, Naples, Italy; ^2^ Unit of Epidemiology, National Cancer Institute, G. Pascale Foundation, Naples, Italy; ^3^ Lester and Sue Smith Breast Center, Baylor College of Medicine, Houston, TX, USA; ^4^ Breast Unit, National Cancer Institute, G. Pascale Foundation, Naples, Italy; ^5^ Clinical Nutrition and Risk Factor Modification Centre, St. Michael’s Hospital, Toronto, Canada; ^6^ Department of Gastroenterology, Endocrinology and Surgery, University of Naples Federico II, Naples, Italy

**Keywords:** breast cancer, obesity, diabetes, combined variables, outcome

## Abstract

**Background:**

Previous studies suggested that obesity and diabetes were correlated with breast cancer outcome. The aim of the present study was to investigate the prognostic effect of obesity and diabetes on the outcome of early breast cancer patients.

**Materials and Methods:**

Overall, 841 early breast cancer patients were prospectively enrolled between January 2009 and December 2013. Study population was divided into four groups: (1) patients without obesity or diabetes; (2) patients with only diabetes; (3) patients with only obesity; and (4) patients with both diabetes and obesity. Categorical variables were analyzed by the chi-square test and survival data by the log-rank test.

**Results:**

At diagnosis, obese and diabetic patients were more likely to be older (*p* < 0.0001) and post-menopausal (*p* < 0.0001) and to have a tumor larger than 2 cm (*p* < 0.0001) than patients in groups 1–3. At univariate analyses, obese and diabetic patients had a worse disease-free survival (*p* = 0.01) and overall survival (*p* = 0.001) than did patients without obesity and diabetes. At multivariate analyses, the co-presence of obesity and diabetes was an independent prognostic factor for disease-free survival (hazard ratio=2.62, 95% CI 1.23–5.60) but not for overall survival.

**Conclusions:**

At diagnosis, patients with obesity and diabetes were older, had larger tumors and a worse outcome compared to patients without obesity or diabetes. These data suggest that metabolic health influences the prognosis of patients affected by early breast cancer.

## INTRODUCTION

The latest World Health Organization (WHO) report estimated breast cancer (BC) incidence in westernized countries at 89.7 per 100,000 women, which makes it the most common cancer among women [1]. The WHO estimated that, in 2014, 422 million adults were affected by diabetes, with a prevalence of 8.5% [2], and more than half a billion people were affected by obesity (body mass index [BMI] ≥ 30 kg/m^2^), with a global prevalence of 15% in women older than 18 years [3]. Diabetes and obesity affect both the BC phenotype [4] and the prognosis of patients [5]. Metabolic health is currently a major issue in daily oncological practice because weight gain, and elevated blood levels of glucose [6], insulin [6], triglycerides [6, 7] and cholesterol [7] are common side effects of adjuvant treatment [7–11]. To date, few studies have analyzed the impact of BMI and diabetes on the outcome of breast cancer patients [12, 13]. The aim of the present prospective trial was to investigate the association between diabetes, obesity and the outcome in patients affected by early BC, in a Mediterranean population.

## RESULTS

### Demographic, clinical and pathologic characteristics

A total of 841 early breast cancer patients: 536 (64%) without diabetes or obesity, 231 (27%) with obesity alone, 34 (4%) with diabetes alone, and 40 with diabetes and obesity (5%) were enrolled in this study. The baseline clinical and pathologic characteristics of the patients in each study group are summarized in Table 1. At diagnosis, women with diabetes and obesity versus the only diabetes, only obesity, and no obesity and no diabetes patients, were more likely to be older (mean age ± SD:66.3 ± 7.8, 62.0 ± 10.6, 58.9 ± 11.4 and 52.3 ± 12.3, respectively, *p* < 0.0001), to be post-menopausal (postmenopausal rate: 98%, 82 %, 77 % and 50%, respectively, *p* < 0.0001), and to have larger tumors (T1 rate: 37%, 47%, 49% and 61%, respectively, *p* < 0.0001). No statistically significant differences were found in nodal status, tumor grade, stage, distribution of histological BC subtypes or type of neo- or adjuvant therapy administered in the four study groups.

**Table 1:** Distribution of patients and clinical-pathological characteristics

	No Diabetes and No obesity (*n* = 536)	Only Obesity (*n* = 231)	Only Diabetes (*n* = 34)	Diabetes AND Obesity (*n* = 40)	*p*-value
**Characteristics**					
**Age at diagnosis (mean ±SD)**	52.3 ±12.3	58.9±11.4	62.0±10.6	66.3±7.8	**< 0.0001**
Age					**< 0.0001**
≤ 55 yrs	341 (64)	80 (35)	11 (32)	4 (10)	
> 55 yrs	195 (36)	151 (65)	23 (68)	36 (90)	
**Menopause**					**< 0.0001**
Post-menopause	268 (50)	177 (77)	28 (82)	39 (98)	
Pre-menopause	268 (50)	54 (23)	6 (18)	1 (2.)	
**Stage**					0.06
I and IIA	203 (39)	65 (30)	12 (38)	11 (29)	
IIB, IIIA-IIIC	153 (30)	64 (29)	10 (31)	14 (37)	
**Grading**					0.25
1	26 (5)	12 (5)	2 (6)	3 (8)	
2	230 (44)	80 (36)	17 (50)	12 (32)	
3	268 (51)	134 (59)	15 (44)	23 (60)	
**N**					0.09
N0	286 (55)	108 (49)	22 (65)	22 (58)	
N1	161 (31)	62 (28)	8 (23)	9 (24)	
N2	55 (10)	35 (16)	2 (6)	7 (18)	
N3	22 (4)	17 (7)	2 (6)	0	
**T**					**0.001**
T1	318 (61)	111 (49)	15 (47)	15 (37)	
T2	181 (35)	101 (45)	12 (37)	23 (58)	
T3-T4	24 (4)	14 (6)	5 (16)	2 (5)	
**Type of therapy administered**					0.2
No adjuvant therapy	31 (6)	21 (10)	3 (9)	3 (8)	
Neo- or adjuvant CT ± HT	82 (17)	25 (12)	3 (9)	2 (5)	
Neo- or adjuvant HT only	381 (77)	165 (78)	26 (82)	32 (87)	
Molecular subtypes^*^					0.9
Luminal A like	203 (40)	86 (38)	13 (38)	14 (37)	
Luminal B like Her2 neg.	152 (30)	70 (31)	11 (32)	13 (34)	
Luminal B like Her2 pos.	63 (12)	25 (11)	5 (15)	6 (16)	
Her2 positive	28 (5)	12 (5)	0	2 (5)	
Triple negative	66 (13)	34 (15)	5 (15)	3 (8)	

CT: chemotherapy; HT: hormone therapy; ^*^St Gallen categorization 2013: Luminal A like: er^+^;pgr>=20; Her2-; ki67<20; Luminal B like Her2 neg.: er^+^;pgr<20 or ki67>=20; Her2-; Luminal B like Her2 pos.: er^+^;pgr^+^; Her2^+^; ki67 any; Her2positive: er-;pgr-; Her2^+^; ki67 any ; Triple negative: er-;pgr-; Her2-; ki67 any.

### Survival analysis

At a median follow-up of 58.9 months, 137 (14.6%) patients experienced tumor recurrences and 67 (7.4%) patients died from their disease. 5-year DFS and OS rates were 85.5% and 92.8%, respectively. Interestingly, at univariate analysis, by pairwise comparison by log-rank test, diabetic and obese women were more likely to relapse and die from breast cancer than patients without obesity or diabetes. In detail, DFS rates were 72.5% vs 86%; *p* = 0.01 and OS rates were 79.5% vs. 93.4% *p* = 0.001 in diabetic and obese patients versus patients without either of these two conditions. No difference in terms of pairwise comparison was observed for DFS and OS in patients with only obesity or only diabetes vs patients with no obesity and no diabetes (*p* = 0.4 and *p* = 0.8, respectively).

**Figure 1:**
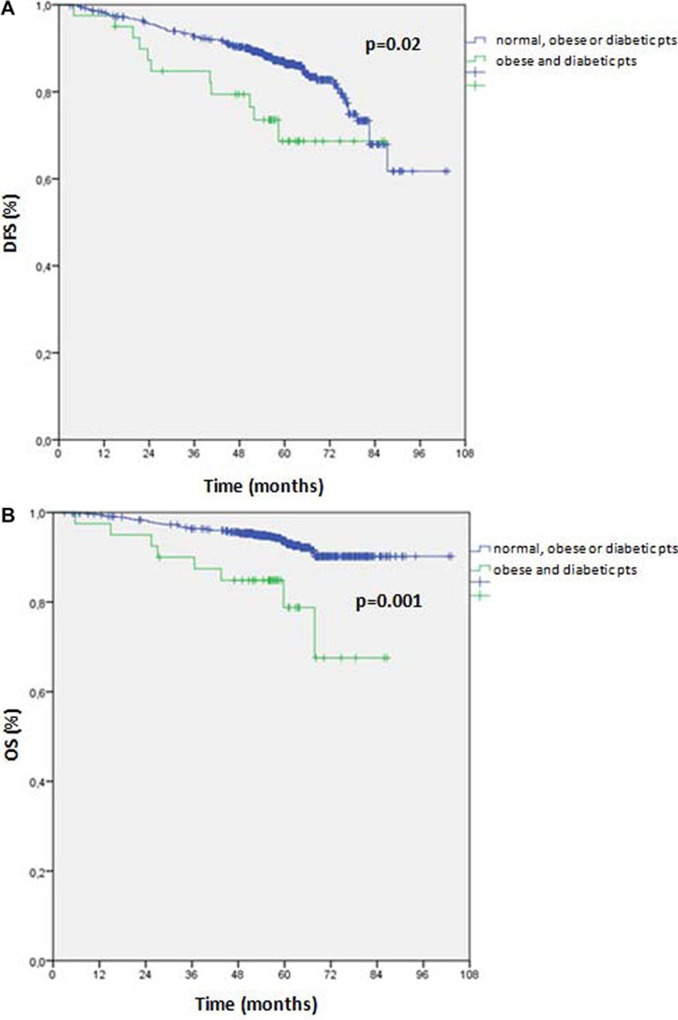
(**A**) Disease free survival (DFS) according to the co-presence of obesity and diabetes (obese and diabetic patients) versus the absence of both or the presence of only one of the two diseases (normal, obese or diabetic patients). (**B**) Overall survival (OS) according to the co-presence of obesity and diabetes (diabetes and obesity patients) versus the absence of both or the presence of only one of the two diseases (normal, obese or diabetic patients). Abbreviations: pts = patients.

In exploratory analyses, we analyzed DFS and OS rates according to the co-presence of obesity and diabetes (obese and diabetic patients) versus the absence of both or the presence of only one of the two diseases (normal, obese or diabetic patients) (Figure 1A and 1B). Interestingly, patients with obesity and diabetes had worse DFS and OS versus patients without either or with only one of these conditions. In detail, DFS rates were 85.3% vs. 72.5% (*p* = 0.02) and OS rates were 93.3% vs. 80.0% (*p* = 0.001), in patients without obesity or diabetes or with only obesity or only diabetes vs. patients with obesity and diabetes, respectively.

At univariate analyses, tumor stage, molecular subtype and type of adjuvant therapy received were also significantly associated to DFS Table 2. In detail, DFS rates were 90.7% vs. 75.1% in patients with Stage I & IIA vs. Stage IIB, IIIA-IIIC BC (*p* < 0.0001); 88.3% vs. 87.2% vs. 84.0% vs. 60.5% vs. 80.8% in patients with luminal A-like vs. luminal B-like Her 2-negative vs. luminal B-like Her 2 positive vs. HER2 positive vs. triple negative BC (*p* < 0.0001) and 87.9% vs. 77. 5% vs. 80.8% in patients receiving no adjuvant therapy vs. neo- or adjuvant CHT and HT vs. neo- or adjuvant HT only (*p* < 0.002). For OS, the variables significant at univariate analyses were patients' age, tumor stage, molecular subtype and type of neo- or adjuvant therapy received. Overall survival rates were 95.9% vs. 86.5% in patients younger than 55 vs older than 55 years (*p* < 0.0001); 95.9% vs. 86.5% in patients with Stage I and IIA vs. Stage IIB, IIIA-IIIC BC (*p* < 0.0001); 93.5% vs. 94.8% vs. 92.3% vs. 86.5% vs. 86.2% in patients with luminal A-like vs. luminal B-like Her 2-negative vs. luminal B-like Her 2-positive vs. HER2-positive vs. triple negative BC (*p* = 0.02); and 94% vs. 90.6% vs. 86.2% in patients not receiving adjuvant therapy vs. neo- or adjuvant CT and HT vs. neo- or adjuvant HT only (*p* = 0.008).

**Table 2:** Disease-free survival and overall survival results: Univariate analysis

Variable	DFS			OS		
	No. of Events	%	Log rank^*^ *p*-value	No. of Events	%	Log rank *p*-value
**Combined Diabetes & Obesity**			**0.07**			**0.008**
No Diabetes and No Obesity	74	*86.0*		34	*93.4*	
Only Obesity	38	*83.3*	0.4	16	*92.7*	0.7
Only Diabetes	4	*88.2*	0.8	3	*90.6*	0.5
Diabetes AND Obesity	11	*72.5*	**0.01**	8	*79.5*	**0.001**
**Age**						
≤ 55 yrs	64	*86.5*	0.2	20	*95.9*	**< 0.0001**
> 55 yrs	72	*93.6*		47	*89.5*	
**Stage**						
I & IIA	55	*90.7*	**< 0.0001**	25	*95.9*	**< 0.0001**
IIB, IIIA - IIIC	70	*75.1*		39	*86.5*	
Molecular subtypes^*^			**< 0.0001**			**0.02**
Luminal A like	45	*88.3*		25	*93.5*	
Luminal B like Her2 neg.	33	*87.2*		13	*94.8*	
Luminal B like Her2 pos.	17	*84.0*		8	*92.3*	
Her2 positive	15	*60.5*		5	*86.5*	
Triple negative	23	*80.8*		15	*86.2*	
**Type of therapy use**			**0.002**			**0.008**
No adjuvant therapy	78	*87.9*		38	*94.0*	
Neo- or adjuvant CT ±HT	32	*77.5*		13	*90.6*	
Neo- or adjuvant HT only	23	*80.8*		15	*86.2*	

^*^Pairwise comparison using a log-rank test.

A multivariate Cox regression analyses, adjusted for the variables that were significant at univariate analyses, showed that the co-presence of diabetes and obesity had an independent prognostic value for DFS (HR = 2.62, 95% CI 1.23–5.60, *p* = 0.01) but not for OS (HR = 2.52; 95% CI 0.97–6.58, *p* = 0.058) (Table 3).

**Table 3:** Adjusted Cox multivariate analysis of breast cancer risk for the combination of diabetes and obesity

	DFS			OS		
	HR*	95% CI	*p*-value	HR	95% CI	*p*-value
No diabetes and no obesity	1		0.06^**^	1		0.08^**^
Only obesity	0.96	0.59–1.56	0.8	0.66	0.30–1.47	0.3
Only diabetes	0.92	0.28–2.99	0.9	1.67	0.48–5.75	0.4
Diabetes and obesity	**2.62**	**1.23–5.60**	**0.01**	2.52	0.97–6.57	0.058

^*^Adjusted for terms of age (continuous), Stage (IA & IIA; IIB, IIIA & IIIB), Molecular subtypes (Luminal A; Luminal B; Triple positive; Her2pos; Triple negative) and Therapy (No therapy/ Neoadj. or adj. CT ± HT/ Neoadj. or adj. HT only) ^**^Wald Test.

## DISCUSSION

Here we demonstrate that obesity and diabetes are independent prognostic factors for DFS in patients affected by early breast cancer treated with standard neo- or adjuvant therapy. The risk of cancer recurrence was approximately three times higher in patients with diabetes and obesity than in patients who were neither obese nor diabetic. Obese and diabetic patients were also more likely to have larger tumors and to be postmenopausal. However, the distributions of tumor grade, neo- or adjuvant therapies and tumor molecular subtypes (based on IHC classification) were similar across the study subgroups.

It is unclear whether diabetes increases breast cancer–specific mortality. Compared with their non-diabetic counterparts, patients with breast cancer and pre-existing diabetes have been described to present with more advanced breast cancer at diagnosis and to receive less aggressive treatments [14–16] given their underlying comorbidities and the perceived risk of greater chemotherapy-related toxicity. The diabetic patients included in our study had larger tumors than did their not diabetic counterparts. However, treatment choices in our patient population were not affected by the preexisting diagnosis of diabetes and the presence of this disease alone did not change the outcome of patients.

Three meta-analyses have recently examined the relationship between BMI and survival in large numbers of patients [17–19]. Overall, obesity was found to be associated with an increased risk of recurrence and death of approximately 35% to 40% after adjusting for tumor-related variables, regardless of menopausal or hormone receptor status [17–19]. Consistently, Ligibel et al. [20] showed that each five-unit increase in BMI was associated with an increase in the risk of cancer recurrence and death, or of death alone, of approximately 8%. Differently from these data, we did not find any significant association between the weight of patients and breast cancer outcome. Discrepancies between our study and other studies may be due to differences in the length of follow-up and in the patient populations analyzed. Moreover, we found that 48% of our patients with BMI < 30, were overweight (BMI > 25 but ≤ 30) and this may have affected the overall prognosis of non obese patients. It is possible that underlying insulin resistance and diabetes may have further worsened prognoses in obese patients included in the afore-mentioned meta-analyses [17–19]. Neither glucose nor insulin level was considered in many of the previously reported trials included in the considered. [17, 19]

Although, neither diabetes nor obesity affected the outcome of our patients, DFS was significantly worse in patients with these two conditions than in patients without them. This result was unrelated to tumor stage, tumor subtype, age and type of neo- or adjuvant therapy received. Our data suggest that diabetes and obesity alone does not necessarily predict metabolic health (defined as no components of the insulin resistance syndrome) and patients who are more metabolically unhealthy (such as obese patients with diabetes) may experience increased breast cancer recurrence.

Our study and other studies support the hypothesis that the detrimental outcome in obese women results mainly from either the presence of insulin resistance and/or diabetes. In fact, Contiero et al. [21] (2013), in a retrospective investigation of 1261 women previously treated for breast cancer (stages I-III), did not find a significant association between BMI ≥ 25 and death from any cause (HR = 1.6, CI 0.88–1.53) irrespective of blood glucose levels. Larsen et al. [22] (2015) in a study of 1,250 postmenopausal breast cancer patients did not find a significant association between overweight or obesity and risk of death from all causes (HR = 0.95, CI 0.72–1.22; HR = 1.09, CI 0.78–1.51, respectively) when diabetes was not included in the analyses. Finally, Herlevic et al. [23] (2015) in a retrospective study of 523 patients with invasive breast cancer did not find a significant association of overweight or obesity alone with OS (*p* = 0.49) or DFS (*p* = 0.33).

Obesity and diabetes are both associated with peripheral tissue insulin resistance, which results in an increase of insulin levels to overcome this peripheral resistance. Insulin, and the insulin like growth factor 1 (IGF1), increase estrogen levels by reducing the concentration of the sex hormone binding protein [24–26] and by enhancing the expression of the aromatase in adipose tissue [27]. Moreover, cross-talk was demonstrated between the IGF1R and HER2 pathways in HER2-positive BC [28, 29]. In pre-clinical BC murine models, diet-induced obesity caused tumor progression by enhancing mesenchymal cell lines and epithelial-to-mesenchymal transition (EMT) markers, while caloric restriction was associated with both suppression of mesenchymal and epithelial cell lines, and inhibition of the EMT [30]. In human mammary epithelial cells, the EMT was also associated with overexpression of the IGF1 receptor [31]. Therefore, the increase in insulin and IGF1 levels, present in both obese and diabetic patients [32], may facilitate the EMT and tumor progression.

Our study has some limitations. The patient cohort was relatively homogeneous in terms of race, education and social status (most patients were well educated, and employed or retired), and thus results may not be generalized to all early breast cancer survivors in other geographic areas. Furthermore, we investigated fasting glucose and have no data on other potentially relevant markers of glucose metabolism and insulin resistance, such as baseline insulin and insulin-like growth factor-I. Importantly, our sample size is relatively modest and, although we adjusted survival analyses for known prognostic factors, it is possible that other factors could influence the relationship between obesity, diabetes and prognosis. Consequently, our data should be considered “hypothesis-generating” and need further validation.

Our study has several strengths: it is a prospective, bi-institutional study; the pre-treatment fasting serum glucose levels of each patient were analyzed by the central laboratory of the participating institute; and, we evaluated a more comprehensive information on patients' metabolic profile by analyzing together both body weight and glucose levels. In conclusion, we report novel evidence in support of a link between obesity and diabetes and breast cancer outcome. Indeed, we show that diabetes combined with obesity affects the outcome of early breast cancer, which suggests that the impairment of metabolic health may be associated to worsening patient prognosis. These findings represent a major issue in oncology daily practice. Based on these findings, improving body weight and blood glucose while avoiding type 2 diabetes through lifestyle changes including diet and exercise is expected to improve survival in people with breast cancer.

## MATERIALS AND METHODS

### Study population and laboratory assays

Eight-hundred and forty-one early breast cancer patients consecutively treated at the National Cancer Institute “G. Pascale Foundation” and at the University Hospital of Naples Federico II, between January 2009 and December 2013 were included in the present study. For each patient we recorded body weight and height, BMI, fasting plasma glucose levels and tumor characteristics, namely, tumor size (T), nodal status (N), stage, estrogen receptor (ER) and progesterone receptor (PgR expression, grading (G), HER2/neu status and tumor proliferation measured by ki67 labeling. According to the American Society of Clinical Oncology-College of American Pathologists guidelines, ER and PgR status were assessed by immunohistochemistry (IHC): the cut-off value used to distinguish ER/PgR “positive” from ER/PgR “negative” was ≥ 1% ER/PR-positive tumor cells [33], while HER 2 expression was considered positive when, within an area of tumor that amounts to > 10% of contiguous and homogeneous tumor cells, there was evidence of protein overexpression at IHC or gene amplification (HER2 copy number or HER2/CEP17 ratio by *in situ* hybridization based on counting at least 20 cells within the area) [34].

The proliferative index Ki67 was assessed according the Recommendations of the International Ki67 in Breast Cancer Working Group [35] and defined as the percentage, at IHC, of immune-reactive tumor cells out of the total number of cells. The percentage of positive cells per case was scored into 2 groups: “low”: < 20% (low proliferative activity) and “high”: ≥ 20% (high proliferative activity). The molecular subtypes of BC were identified and categorized according to the 13^th^ St Gallen International Breast Cancer Conference (2013) Expert Panel [36]. The BMI, calculated as weight divided by height squared, was stratified according to the WHO classification (≤ 30 kg/m^2^ = overweight/normal; > 30 kg/m^2^ = obese). Fasting plasma glucose levels were assessed from blood samples according to the NCEP ATP III criteria [37] and categorized as follows: normoglycemia < 126 mg/dl; diabetes ≥ 126 mg/dl, according to the latest American Diabetes Association guidelines [38]. Women with normoglycemia at the time of the blood test who reported taking diabetic medications were allocated to the diabetes category. Based on the presence of obesity and diabetes, patients were divided in four subgroups: patients without obesity or diabetes; patients with only diabetes; patients with only obesity; and patients with both diabetes and obesity.

The study was approved by the Institutional Review Board of the University of Naples Federico II (IRB approval # 743/15) and participants provided written informed consent to participate. The records and data of patients were anonymized and de-identified prior to analysis.

### Statistical analyses

Descriptive statistics for categorical data are provided in terms of absolute and relative frequencies. The non-normally distributed continuous variable age is described by mean and standard deviation. Associations between the combination of study groups and patients or tumor characteristics were evaluated using the Kruskal-Wallis test for age, and the chi-square test for all other categorical variables: menopausal status, stage, grading, nodal stage, tumor size, neoadjuvant and adjuvant therapy, molecular subtypes and patient-status. The outcome of patients was analyzed in terms of both disease-free survival (DFS: with local, contralateral and distant disease recurrence, and secondary primary tumors and death from any cause defined as the event) and overall survival (OS: with death from any cause defined as the event). Time-to-event data (median follow-up 58.9 months, range 1–108 months) were analyzed using the Kaplan-Meier method and summarized using medians, 95% confidence intervals (CI), and survival curves. Study groups were compared using log-rank tests. To evaluate whether the co-presence of diabetes and obesity constitutes an independent prognostic factor, we used Cox proportional hazards regression models adjusted for age (continuous), stage (I and IIA; IIB and IIIA-IIIC), molecular subtypes (Luminal A-like; Luminal B-like Her2-negative; Luminal B-like Her2-positive; Her2-positive: ER/PgR absent; triple negative) and therapy (no adjuvant therapy/neo- or adjuvant chemotherapy (CHT) ± hormone therapy (HT)/neo- or adjuvant HT only). The category “No obesity and No diabetes” served as the reference group for the calculations of hazard ratios (HR) and 95% CI. All statistical tests were two-sided, and *p* values below 0.05 were considered statistically significant (no adjustment of significance levels for multiple testing). Statistical analyses were performed using IBM^®^ SPSS^®^ Statistics, version 23 (IBM Corp., Armonk, NY, USA)
